# Geptop 2.0: An Updated, More Precise, and Faster Geptop Server for Identification of Prokaryotic Essential Genes

**DOI:** 10.3389/fmicb.2019.01236

**Published:** 2019-06-04

**Authors:** Qing-Feng Wen, Shuo Liu, Chuan Dong, Hai-Xia Guo, Yi-Zhou Gao, Feng-Biao Guo

**Affiliations:** School of Life Sciences and Technology, Center for Informational Biology, University of Electronic Science and Technology of China, Chengdu, China

**Keywords:** essential genes, prediction, prokaryotes, software, bioinformatics

## Abstract

Geptop has performed effectively in the identification of prokaryotic essential genes since its first release in 2013. It estimates gene essentiality for prokaryotes based on orthology and phylogeny. Genome-scale essentiality data of more prokaryotic species are available, and the information has been collected into public essential gene repositories such as DEG and OGEE. A faster and more accurate toolkit is needed to meet the increasing prokaryotic genome data. We updated Geptop by supplementing more validated essentiality data into reference set (from 19 to 37 species), and introducing multi-process technology to accelerate the computing speed. Compared with Geptop 1.0 and other gene essentiality prediction models, Geptop 2.0 can generate more stable predictions and finish the computation in a shorter time. The software is available both as an online server and a downloadable standalone application. We hope that the improved Geptop 2.0 will facilitate researches in gene essentiality and the development of novel antibacterial drugs. The gene essentiality prediction tool is available at http://cefg.uestc.cn/geptop.

## Introduction

Essential genes are critical for the survival and development of organisms (Mushegian and Koonin, 1996). In dozens of prokaryotes, genome-scale essentiality data have been determined by various experimental methods and these information has been stored in online databases such as DEG (Luo et al., 2014) and OGEE (Chen et al., 2017). Studies on bacterial essential genes are helpful in understanding the essence of life (Rancati et al., 2018) and screening potential drug targets to treat pathogenic diseases (Dickerson et al., 2011).

Due to the cost and difficulties of experiments, computational identification of essential genes presents an important alternative approach (Mobegi et al., 2017). Features including evolutionary conservation (Nigatu et al., 2017; Dilucca et al., 2018), domain information (Lu et al., 2015), network topology (Jeong et al., 2001; Zhang et al., 2016; Karthik et al., 2018; Li et al., 2019), function (Lei and Yang, 2018), and expression level (Dong et al., 2018) are used in predicting gene essentiality via the approaches of bioinformatics. Based on this, many models were developed to implement gene essentiality prediction (Fan et al., 2017; Mobegi et al., 2017; Li et al., 2019; Zhang et al., 2019).

There are a few online tools to automatically predict bacterial essential genes. CEG_Match was developed to select name-known essential genes based on gene function information (Ye et al., 2013). Essential Gene Prediction (EGP) is a machine-learning-based method using only sequence compositional features (Ning et al., 2014). We utilized another machine learning algorithm (SVM) with features obtained by homology mapping to predict gene essentiality (Hua et al., 2016). The BLAST tool in DEG can be utilized to perform homolog search against essential gene set (Luo et al., 2014).

In 2013, we released Geptop to predict essential genes of sequenced bacterial genomes. It uses the reciprocal best hit (RBH) method to determine orthology, and the composition vector (CV) method to weight the contributions of each reference genome (Qi et al., 2004; Wei et al., 2013). Since the release of Geptop 1.0, it has become the most widely used computational tool for predicting bacterial essential genes, in large part due to its high accuracy and the availability as Web server (Gupta et al., 2016; Gao et al., 2017; Nigatu et al., 2017; Peng et al., 2017; Rancati et al., 2018). When using only three genomes as reference set, Geptop is competitive with other integrative methods, and its superiority becomes more pronounced when 18 genomes are used as the reference set (Wei et al., 2013). The excellent performance of Geptop depends on our intrinsic definition of orthology using RBH and the method of balancing the weights of various reference genomes according to their phylogenetic distances.

Since our release of Geptop 1.0, the number of genomes with essential gene data has increased significantly (Luo et al., 2014; Chen et al., 2017). In addition, the Python package contains a multiprocessing module that can execute multiprogramming computation, which obviously makes the process of calculating faster. In light of the progress in data and technology, we were inspired to update Geptop.

## Methods

Information about gene essentiality was obtained from DEG or OGEE, and the complete protein coding sequences of all 40 bacteria were acquired from GenBank. Detailed information is displayed in Supplementary Table S1. Similar with that in Geptop 1.0 (Wei et al., 2013), each of the 40 species was used as the test set, and the other 39 proteomes being used as the reference set, separately. After obtaining the 40 area under the curve (AUC) scores calculated based on the real essentiality annotation and the predicted essentiality score, we only selected those genomes whose AUC scores were higher than 0.60 as the final reference set. And three genomes got the AUC scores lower than 0.60. Indeed, essentiality data of the three eliminated genomes may have significant biases (Wei et al., 2013; Cheng et al., 2014).

Geptop 1.0 used the method of geometric mean to combine the contribution of each reference genome to obtain the essentiality score of inquiry gene. When the orthologous gene in one reference proteome is non-essential or the inquiry gene has no ortholog, we need to neglect the contribution of this reference genome and only consider those reference genomes that contain essential orthologs. This manner of dealing with this issue generates reasonable essentiality score; however, the original equation for implementing this operation in Geptop 1.0 may be difficult to understand.

In the new implementation of Geptop 2.0, to define the gene essentiality score *S_i_* for gene *i*, we changed the cumulative formula as follows:

(1) $$S_{i}=\frac{1}{N}\sum_{j=1}^{N}\frac{M_{ij}}{D_{j}}$$

where *j* is the *j^th^* proteome in the reference set, *N* is the count of all reference proteomes, *M* is the mapping score. *M* is two-value-variables “1” and “0.” “1” means the ortholog of a query gene is essential in the reference genome whereas “0” means that a query gene has not any orthologs or the ortholog is non-essential. *D_j_* is the evolutionary distance between the query proteome and the *j^th^* reference proteome. It can be calculated by the CV method (Qi et al., 2004). When *D* = 0, which means that the query genome and reference genome are the same, we set *D* to 0.01 to avoid division by zero. After obtaining the essentiality scores for all genes in the query genome, we use the following formula to normalize the result:

(2) $$S_{final}=\frac{S_{i}-Min}{Max-Min}$$

where *S_final_* is the final *S* score of gene *i, Min* is the minimum of *S* over all genes in the query proteome, and *Max* is the maximum. *S_final_* ranges from 0 to 1, and the more essential a gene is, the larger the value of *S_final_* will be.

In addition, we utilized the multiprocessing module in Python to increase computational efficiency.

### Performance Assessment

The following indexes were used to assess the performance of the predictor:

$$\mathrm{sensitivity}=\frac{TP}{TP+FN}$$

$$\mathrm{specificity}=\frac{TN}{TN+FP}$$

$$\mathrm{precision}=\frac{TP}{TP+FP}$$

$$F-\mathrm{measure}=\frac{2*sensitivity*precision}{sensitivity+precision}$$

$$\mathrm{MCC}=\frac{TP*TN-FP*FN}{\sqrt{\left(TN+FN)*(TN+FP)*(TP+FN)*(TP+FP\right)}}$$

Here *TP, FN, FP*, and *TN* denote the true positives, false negatives, false positives, and true negatives, respectively. The sensitivity index represents the proportion of essential genes that have been correctly identified, and the specificity index represents the proportion of non-essential genes that have been correctly identified, and the precision index is the probability that the predicted essential genes were indeed essential. The F-measure represents the harmonic mean of precision and sensitivity. The Mathew Correlation Coefficient (MCC) index represents the reliability of the algorithm which ranges from -1 to 1. When *FP* and *FN* are both equal to 0, MCC is equal to 1, meaning that the result of prediction is totally right; conversely, if *FP* and *FN* are both equal to 1, MCC is equal to -1, meaning that the result of prediction is totally wrong.

## Results

### Improved Performance of Geptop 2.0 Than Geptop 1.0 by Cross-Species-Validation

**FIGURE 1:**
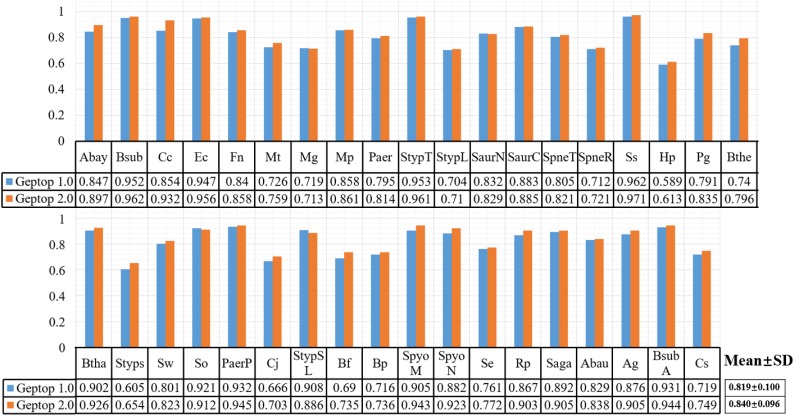
The AUC result of 37 genomes among Geptop 1.0, Geptop 2.0. And the abbreviation of 37 bacteria please refers to Supplementary Table S1.

After choosing 37 genomes with reliable essentiality information and aligning them as reference set in Geptop 2.0, we performed further computation to compare the prediction performance between Geptop 1.0 and 2.0. For this purpose, we ran both versions for each of the 37 genomes and obtained 37 pairs of AUC scores by cross-validations. The results are shown in Figure 1, in which each genome is represented by one column. The average AUC value of Geptop 1.0 among the 37 genomes was about 0.82, whereas that of Geptop 2.0 was higher than 0.84, indicating an improvement of over 2.0%. And the variance in Geptop 2.0 was less than that in Geptop 1.0 among the 37 AUC values. In the case of *Caulobacter crescentus* NA1000 (Cc), the AUC score of Geptop 2.0 was 8.0% higher.

After changing the scoring method as shown in formulae (1) and (2), we reset the default threshold value of the essentiality score to 0.24, meaning that if the essentiality score of one gene is higher than 0.24, then the gene should be predicted as essential gene. Moreover, users may also change this threshold according to their specific requirements to ensure fewer false positives or higher precision.

Besides AUC evaluation, we also utilized the sensitivity, specificity, MCC and F-measure indexes to assess the prediction performance for both versions of Geptop. The complete results are shown in Supplementary Table S2. Except that the average specificity index among 37 species is about 0.9% lower in Geptop 2.0 than that in Geptop 1.0, other averages for indexes in Geptop 2.0 are higher ranging from 1.5 to 4.3%.

### The Advantage of Geptop 2.0 Compared With Other Essential Gene Prediction Models

**FIGURE 2:**
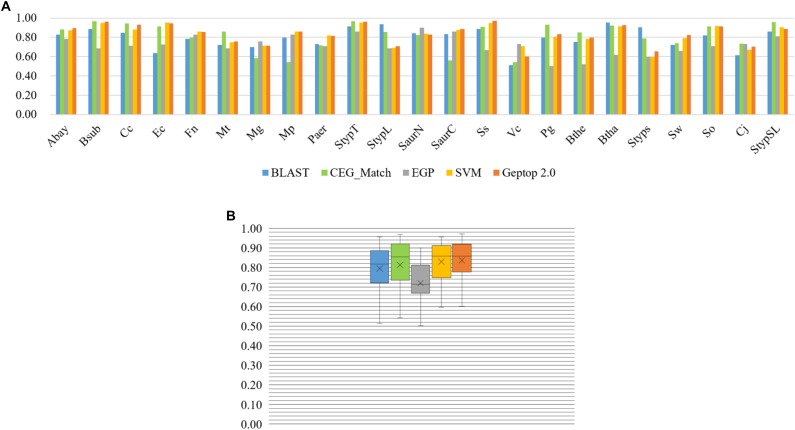
Prediction performance of BLAST tool, CEG_Match, EGP, SVM, and Geptop 2.0 in the 23 genomes. **(A)** AUC scores of the gene essentiality prediction by BLAST tool, CEG_Match, EGP, SVM, and Geptop 2.0, respectively, for each genome. **(B)** Box plot of AUC scores from the prediction of the five tools for the 23 genomes.

By AUC evaluation only, Geptop 2.0 is competitive with other models. We performed prediction in four models introduced above for 23 organisms (Hua et al., 2016; Peng et al., 2017). The result was exhibited in Figure 2. Except EGP, all these models performed well in the 23 organisms. The lowest AUC score among 23 organisms of Geptop 2.0 is still higher than 0.60, and the highest is 0.97, while all other models have some AUC scores lower than 0.60, indicating that the prediction of Geptop 2.0 is reliable enough. Considering the average AUC score of 23 organisms, Geptop 2.0 has the value of 0.84, which is the highest among the five models.

## Discussion

The species distance of query genome with reference genomes can influence the prediction performance, so we calculated the minimal distance between query genome and the 36 reference genomes, then the closest genomes would be removed from reference set. We performed prediction again for the query genome using 35 reference genomes. The 14 species whose AUC scores higher than 0.90 in Geptop 2.0 were selected. Loss of closest species in reference genomes would indeed decrease the performance in most case. However, the difference is slight and the average decrease of AUC is only about 1%. There is even 0.1% increment of AUC for *Salmonella enterica serovar* Typhi Ty2.

**FIGURE 3:**
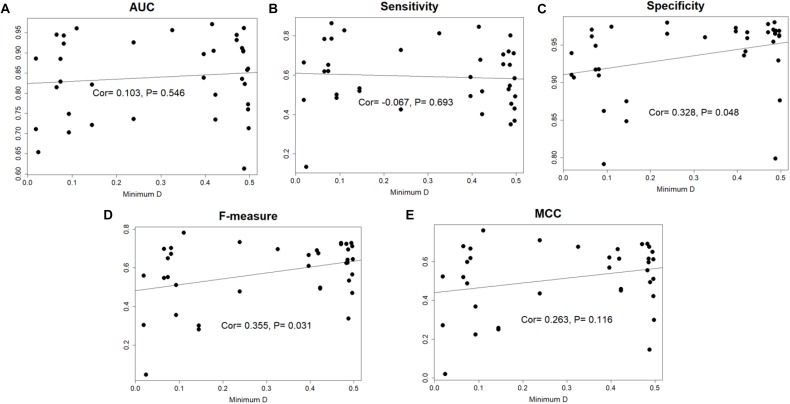
The correlation between minimal species distance and prediction performance among 37 genomes. In each sub-picture, the horizontal axis represents the minimal distance between the query genome and 36 reference genomes. The vertical axis represents the prediction performance estimated by AUC, Sensitivity, Specificity, F-measure and MCC indexes. The “Cor” value in figure is “Pearson correlation coefficient.” **(A)** The correlation between AUC score and minimal species distance. **(B)** The correlation between the sensitivity index and minimal species distance. **(C)** The correlation between the specificity index and minimal species distance. **(D)** The correlation between the F-measure index and minimal species distance. **(E)** The correlation between MCC index and minimal species distance.

In the total 37 genomes, 18 genomes obtained a minimal distance higher than 0.41 and this is quite far species distance. Among these 18 genomes, 8 genomes got an AUC score higher than 0.90, while only one genome got an AUC score lower than 0.70, indicating that the performance of Geptop 2.0 is stable and reliable in the essential genes identification without close reference genomes. If we directly calculate the correlation coefficient between minimal distance and each of the five indexes for the 37 species, it will find there is not significant association (Figure 3). Hence, Geptop’s performance generally relies on the scale of reference genomes and a few special genomes will have trivial influence on it. When there are fewer reference genomes, the prediction accuracy will depend significantly on the reference genome with the smallest distance. However, if we have numerous reference genomes, the effect of smallest evolution distance on prediction accuracy will be significantly weaken and all reference genomes will play a collective effect on the prediction. In fact, a larger number of genomes could also weak the quality bias caused by one or two special reference genomes. Therefore, updating Geptop into the version 2.0 could generate more stable prediction.

## Conclusion

With more reference genomes, Geptop 2.0 can get better performance than Geptop 1.0, and it’s competitive with other gene essentiality prediction models. Despite the limitation in some species, its performance is reliable enough in most species. We are confident that Geptop 2.0 would generate more stable predictions with larger-scale reference set. Besides, we used the multiprocessing module to achieve the multiprogramming computation using a Linux 4 CPU system, ultimately increasing the computation efficiency by more than fourfold. For example, for *Escherichia coli* K12 MG 1655 (4100 genes in total), our server returned prediction results in less than 35 min. The web server and standalone version of Geptop are available at http://cefg.uestc.cn/geptop.

## Data Availability

Publicly available datasets were analyzed in this study. This data can be found here: http://origin.tubic.org/deg/public/index.php.

## Author Contributions

F-BG designed and coordinated this project and revised the manuscript. Q-FW the programmed Geptop 2.0 and revised both the website and software. SL checked the AUC results. Q-FW drafted the manuscript. CD and SL checked the Geptop algorithm. H-XG and Y-ZG took part in the AUC computation. All authors read and approved this manuscript.

## Conflict of Interest Statement

The authors declare that the research was conducted in the absence of any commercial or financial relationships that could be construed as a potential conflict of interest.
