# Supplementary material for: Geptop 2.0: An Updated, More Precise, and Faster Geptop Server for Identification of Prokaryotic Essential Genes
Source: Front Microbiol. 2019 Jun 4;10:1236. doi: 10.3389/fmicb.2019.01236 (PMC6558110; doi:10.3389/fmicb.2019.01236)
Supplement: Supplementary file 1 [file Data_Sheet_1.PDF]

Supplementary Material

Supplementary Table S1: The general information of 40 bacteria used in the work. The AUC value was calculated based on the real essentiality annotation obtained from DEG or OGEE and the predicted essentiality score obtained from Geptop when the all 40 genomes were used as reference set. We only selected those genomes whose AUC scores were higher than 0.60 as the final reference set, and the three eliminated genomes were presented in bold faces.

Species	Abbreviations	Number of Essential Genes	Number of Total Gene	AUC
Acinetobacter baylyi ADP1	Abay	499	3307	0.893
Bacillus subtilis 168	Bsub	271	4175	0.958
Caulobacter crescentus NA1000	Cc	480	3885	0.928
Escherichia coli MG1655	Ec	296	4140	0.946
Francisella novicida U112	Fn	390	1719	0.854
Mycobacterium tuberculosis H37Rv	Mt	612	3907	0.756
Mycoplasma genitalium G37	Mg	379	476	0.714
Mycoplasma pulmonis UAB CTIP	Mp	310	782	0.857
Pseudomonas aeruginosa UCBPP-PA14	Paer	335	5892	0.814
Salmonella enterica serovar Typhi Ty2	StypT	358	4352	0.964
Salmonella enterica serovar Typhimurium LT2	StypL	230	4451	0.719
Staphylococcus aureus N315	SaurN	302	2582	0.836
Staphylococcus aureus NCTC 8325	SaurC	350	2771	0.886

Streptococcus pneumoniae TIGR4	SpneT	111	2105	0.825
Streptococcus pneumoniae R6	SpneR	133	1819	0.722
Streptococcus sanguinis SK36	Ss	218	2270	0.970
***Vibrio cholerae* N16961**	**Vc**	**779**	**3693**	**0.589**
***Haemophilus influenzae* Rd KW20**	**Hi**	**642**	**1625**	**0.561**
Helicobacter pylori 26695	Hp	323	1486	0.608
Porphyromonas gingivalis ATCC 33277	Pg	463	2089	0.838
Bacteroides thetaiotaomicron VPI-5482	Bthe	325	4778	0.792
Burkholderia thailandensis E264	Btha	406	5632	0.923
Salmonella enterica subsp. enterica serovar Typhimurium str. 14028S	Styps	105	5315	0.651
Sphingomonas wittichii RW1	Sw	535	4850	0.824
Shewanella oneidensis MR-1	So	402	4065	0.922
Pseudomonas aeruginosa PAO1	PaerP	336	5572	0.946
Campylobacter jejuni subsp. jejuni NCTC 11168 = ATCC 700819	Cj	228	1577	0.700
Salmonella enterica serovar Typhimurium SL1344	StypSL	353	4446	0.885
Bacteroides fragilis 638R	Bf	547	4290	0.731
Burkholderia pseudomallei K96243	Bp	505	5727	0.732
Streptococcus pyogenes MGAS5448	SpyoM	227	1865	0.944

Streptococcus pyogenes NZ131	SpyoN	241	1700	0.924
Synechococcus elongatus PCC 7942	Se	680	2612	0.774
Rhodopseudomonas palustris CGA009	Rp	544	4878	0.902
Streptococcus agalactiae A909	Saga	309	2054	0.899
Acinetobacter baumannii ATCC 17978	Abau	606	3351	0.839
Agrobacterium f	Ag	361	5355	0.905
Brevundimonas subvibrioides ATCC 15264	BsubA	412	3298	0.942
***Bacillus thuringiensis* BMB171**	**Bthu**	**490**	**5360**	**0.563**
Campylobacter jejuni subsp. jejuni 81-176	Cs	384	1653	0.741

Supplementary Table S2: The comparison of interspecies prediction between Geptop 1.0 and Geptop 2.0 among 37 species by AUC, sensitivity, specificity, F-measure and MCC indexes.

	**AUC**		**Sensitivity**		**Specificity**	
**Species**	**Geptop 1.0**	**Geptop 2.0**	**Geptop 1.0**	**Geptop 2.0**	**Geptop 1.0**	**Geptop 2.0**
Abay	0.847	0.897	0.515	0.589	0.978	0.968
Bsub	0.952	0.962	0.760	0.801	0.973	0.965
Cc	0.854	0.932	0.531	0.654	0.984	0.978
Ec	0.947	0.956	0.834	0.811	0.966	0.960

Fn	0.840	0.858	0.636	0.710	0.949	0.929
Mt	0.726	0.759	0.322	0.368	0.973	0.963
Mg	0.719	0.713	0.462	0.491	0.887	0.876
Mp	0.858	0.861	0.584	0.584	0.964	0.962
Paer	0.795	0.814	0.496	0.618	0.970	0.961
StypT	0.953	0.961	0.791	0.827	0.973	0.974
StypL	0.704	0.710	0.448	0.474	0.927	0.910
SaurN	0.832	0.829	0.613	0.619	0.914	0.917
SaurC	0.883	0.885	0.654	0.651	0.943	0.949
SpneT	0.805	0.821	0.541	0.532	0.880	0.875
SpneR	0.712	0.721	0.541	0.519	0.852	0.848
Ss	0.962	0.971	0.849	0.844	0.942	0.936
Hp	0.589	0.613	0.307	0.350	0.836	0.799
Pg	0.791	0.835	0.486	0.527	0.965	0.954
Bthe	0.740	0.796	0.471	0.517	0.969	0.959
Btha	0.902	0.926	0.606	0.727	0.982	0.980
Styps	0.605	0.654	0.143	0.133	0.902	0.907
Sw	0.801	0.823	0.376	0.454	0.973	0.969
So	0.921	0.912	0.709	0.719	0.981	0.971
PaerP	0.932	0.945	0.768	0.783	0.972	0.970

Cj	0.666	0.703	0.410	0.485	0.844	0.791
StypSL	0.908	0.886	0.819	0.663	0.942	0.939
Bf	0.690	0.735	0.371	0.400	0.976	0.967
Bp	0.716	0.736	0.341	0.426	0.968	0.965
SpyoM	0.905	0.943	0.709	0.863	0.928	0.918
SpyoN	0.882	0.923	0.643	0.784	0.916	0.910
Se	0.761	0.772	0.379	0.429	0.976	0.969
Rp	0.867	0.903	0.528	0.546	0.982	0.980
Saga	0.892	0.905	0.667	0.676	0.953	0.942
Abau	0.829	0.838	0.455	0.493	0.976	0.973
Ag	0.876	0.905	0.551	0.651	0.975	0.969
BsubA	0.931	0.944	0.614	0.704	0.973	0.967
Cs	0.719	0.749	0.414	0.500	0.887	0.862
Average	0.819	0.840	0.550	0.592	0.945	0.936
variance	0.100	0.096	0.163	0.160	0.042	0.048

	**F-measure**		**MCC**	
**Species**	**Geptop 1.0**	**Geptop 2.0**	**Geptop 1.0**	**Geptop 2.0**
Abay	0.599	0.622	0.599	0.622
Bsub	0.689	0.677	0.689	0.677

Cc	0.626	0.692	0.626	0.692
Ec	0.718	0.678	0.718	0.678
Fn	0.632	0.651	0.632	0.651
Mt	0.411	0.422	0.411	0.422
Mg	0.288	0.300	0.288	0.300
Mp	0.616	0.613	0.616	0.613
Paer	0.468	0.521	0.468	0.521
StypT	0.732	0.763	0.732	0.763
StypL	0.286	0.272	0.286	0.272
SaurN	0.476	0.489	0.476	0.489
SaurC	0.584	0.599	0.584	0.599
SpneT	0.269	0.257	0.269	0.257
SpneR	0.268	0.249	0.268	0.249
Ss	0.683	0.665	0.683	0.665
Hp	0.148	0.145	0.148	0.145
Pg	0.548	0.557	0.548	0.557
Bthe	0.463	0.459	0.463	0.459
Btha	0.637	0.711	0.637	0.711
Styps	0.021	0.019	0.021	0.019
Sw	0.442	0.495	0.442	0.495

So	0.731	0.693	0.731	0.693
PaerP	0.679	0.681	0.679	0.681
Cj	0.226	0.224	0.226	0.224
StypSL	0.635	0.523	0.635	0.523
Bf	0.459	0.452	0.459	0.452
Bp	0.372	0.436	0.372	0.436
SpyoM	0.584	0.668	0.584	0.668
SpyoN	0.528	0.618	0.528	0.618
Se	0.487	0.512	0.487	0.512
Rp	0.560	0.617	0.560	0.617
Saga	0.638	0.616	0.638	0.616
Abau	0.548	0.570	0.548	0.570
Ag	0.552	0.598	0.552	0.598
BsubA	0.647	0.691	0.647	0.691
Cs	0.328	0.368	0.328	0.368
Average	0.502	0.517	0.502	0.517
variance	0.173	0.177	0.173	0.177
